# Antibacterial activity of organic crude extract of Camellia sinensis on bi-species cariogenic biofilm

**DOI:** 10.21142/2523-2754-1402-2026-285

**Published:** 2026-04-04

**Authors:** Margarita Requena-Mendizábal, Rocío Bocanegra-Arista, Dora J. Maurtua-Torres, Tania Rosales-Cifuentes, Ingrit E. Collantes-Díaz, Diana C. Flores-León, Freddy R. Valdez-Jurado, Roger Calla-Poma

**Affiliations:** 1 Faculty of Dentistry, National University of San Marcos. Lima, Peru. mrequenam@unmsm.edu.pe, rbocanegraa@unmsm.edu.pe, trosalesc@unmsm.edu.pe, fvaldezj@unmsm.edu.pe, rcallap@unmsm.edu.pe Universidad Nacional Mayor de San Marcos Faculty of Dentistry National University of San Marcos Lima Peru mrequenam@unmsm.edu.pe rbocanegraa@unmsm.edu.pe trosalesc@unmsm.edu.pe fvaldezj@unmsm.edu.pe rcallap@unmsm.edu.pe; 2 Faculty of Science, Cayetano Heredia University. Lima, Peru. dora.maurtua@upch.pe Universidad Peruana Cayetano Heredia Faculty of Science Cayetano Heredia University Lima Peru dora.maurtua@upch.pe; 3 Faculty of Chemical Engineering, National University of Engineering. Lima, Peru. icollantesd@uni.edu.pe Universidad Nacional de Ingeniería Faculty of Chemical Engineering National University of Engineering Lima Peru icollantesd@uni.edu.pe; 4 National Institute of Health. Lima-Peru. dflores@ins.gob.pe National Institute of Health Lima Peru dflores@ins.gob.pe

**Keywords:** biofilm, *Streptococcus mutans*, *Streptococcus gordonii*, dental caries, *Camellia sinensis*, biopelícula, *Streptococcus mutans*, *Streptococcus gordonii*, caries dental, *Camellia sinensis*

## Abstract

**Objective:**

The study aimed to determine the antibacterial inhibitory activity and anti-biofilm effect of the ethanolic crude extract of *Camellia sinensis* leaves on bi-species oral bacterial biofilms.

**Materials and methods:**

An ethanolic crude extract of *Camellia sinensis* leaves was used. *Streptococcus mutans* ATCC 25175 and *Streptococcus gordonii* ATCC 51656 were cultivated and used under anaerobic conditions for biofilm formation. Three groups were formed: *Camellia sinensis* extract, 0.12% chlorhexidine, and dimethyl sulfoxide. The inhibitory effect was determined by using the Kirby-Bauer Disk Diffusion Agar method, measuring the zone of inhibition formed around each of them. Additionally, the minimum inhibitory concentration was identified, considering the concentration where no growth of bacteria was observed. Immunofluorescence analysis was performed to evaluate cell viability in biofilms. Student’s t-test for independent samples was used to compare groups.

**Results:**

A zone of inhibition of 17.4±0.4 mm for *S. mutans*, and 13.1 ± 0.3 mm for *S. gordonii* was produced by *Camellia sinensis*, while chlorhexidine produced 23.6 ± 0.3 mm for *S. mutans*, and 13.2 ± 0.5 mm for *S. gordonii*. The minimum inhibitory concentration was 3.12 mg/dL for *S. mutans* and 1.56 mg/dL for *S. gordonii.* Analysis of cell viability after 48 hours of incubation showed that the *Camellia sinensis* extract reduced the viability of biofilm cells.

**Conclusion:**

Despite the study's limitations, *Camellia sinensis* shows antibacterial activity against multispecies cariogenic biofilms, with sensitivity in the tested bacteria.

## RESUMEN

**Objetivo:**

Determinar la actividad antibacteriana y efecto antibiofilm del extracto crudo etanólico de hojas de *Camellia sinensis* (té verde) sobre biofilm bi-especies.

**Materiales y métodos:**

Se utilizó un extracto etanólico crudo de té verde. Para la formación del biofilm se cultivaron cepas de *Streptococcus mutans* ATCC 25175 y *Streptococcus gordonii* ATCC 51656 bajo condiciones anaeróbicas. Se formaron 3 grupos: extracto de té verde, clorhexidina al 0,12% y dimetilsulfoxido como control negativo. El efecto antibacteriano se determinó mediante el método disco difusión en agar de Kirby-Bauer, midiendo el halo de inhibición formado en cada disco. Además, se identificó la concentración mínima inhibitoria con el método de microdilución. Se realizó el análisis de inmunofluorescencia para evaluar la viabilidad celular en el biofilm. Se utilizó la prueba t de Student para muestras independientes y comparar entre grupos.

**Resultados:**

*Camellia sinensis* generó un halo de inhibición para *S. mutans* de 17,4 ± 0,4 mm y para *S. gordonii* de 13,1 ± 0,3 mm, mientras que la clorhexidina produjo inhibición de 23,6 ± 0,3 mm y 13,2 ± 0,5 mm para *S. mutans* y *S. gordonii*, respectivamente. La concentración mínima inhibitoria fue de 3,12 mg/dl para el *S. mutans* y de 1,56 mg/dl para *S. gordonii*. El análisis de viabilidad celular con inmunofluorescencia mostró que, tras 48 horas de incubación, el extracto de *Camellia sinensis* redujo la viabilidad de las células del biofilm.

**Conclusiones:**

Pese a las limitaciones del estudio, se puede afirmar que el extracto de té verde presenta actividad antibacteriana frente al biofilm cariogénico multiespecies.

## INTRODUCTION

Nowadays, it is estimated that around 2.500 million people in the world suffer from untreated dental caries and 743 million from severe periodontitis ^1^. The high prevalence of these diseases represents a predictive factor for oral and general health, especially at early ages, eventually affecting people’s well-being ^2^. From a physiological position, the alteration of host response, virulent plaque biofilm, age-related immunological deterioration, and inflammation are responsible for degenerative changes in cells and tissues, increasing the risk of oral diseases ^3^. 

These oral diseases are modulated by the dysbiosis of different microorganisms with *Streptococcus mutans* and *Streptococcus gordonii* being the predominant species. *S. gordonii* is a primary colonizer that facilitates adhesion to the tooth surface and provides binding sites for subsequent colonizers ^4^. These bacteria are known for their ability to form biofilm and are crucial in dental caries development, and periodontal problems ^5^^,^^6^. On the other hand, *S. mutans* can outcompete *S. gordonii* under acidic conditions, contributing to the pathogenic shift in the microbial community. This ecological interplay, characterized by competition, special organization and signaling, makes the bi-species model particularly relevant for studying antimicrobial interventions aimed at disrupting early biofilm development. 

Different treatment strategies have focused on minimizing the impact of caries among the population through promoting good oral hygiene practices and the regulation of cariogenic food consumption ^7^. However, this is not enough as oral diseases morbidity indicators remain high ^1^. 

In recent years, the use of natural products has been promoted as an alternative treatment for diverse oral pathologies that have been demonstrated to be effective with apparent low secondary effects ^8^^,^^9^. Within a wide alternative range, *Camellia sinensis*, is one of the most studied plants due to its versatile pharmacological activity against different microorganisms. This plant is widely used, as an antioxidant, anticarcinogenic, glucose controller, antiviral, and antibacterial, among others ^10^^,^^11^.

Some studies have been conducted on the effectiveness of various *Camellia sinensis* extracts against oral microorganisms related to plaque and dental caries formation, however, some of these studies have been conducted on isolated bacteria ^12^^,^^13^. Moreover, some of them have only studied *S. mutans* oral biofilm but not *S. gordonii*, which plays an important role in the development of bacterial plaque ^14^^-^^16^. 

As is well known, bacterial plaque dysbiosis is one of the primary factors in the development of caried an periodontal diseases ^17^. In this context, it is important to continue researching natural alternatives such as *Camellia sinensis* which have antibacterial properties that can help control oral biofilms. These two species were selected to create a dual-species biofilm model simulating the dynamic interaction between commensal and pathogenic oral streptococci during the early stages of plaque formation.

Using *Camellia sinensis* alongside oral dysbiosis controllers and oral hygiene techniques, and sugar consumption control could reduce the risk of developing oral pathologies. Considering their proven versatile pharmacological activity, affordable production, and lower secondary effects, different *Camellia sinensis* extracts are presented as the ideal alternatives ^15^. They can be used either as alternatives or to complement the strategies against different oral pathologies. 

This study aimed to determine the antibacterial effect of crude *Camellia sinensis* extract on a multispecies oral biofilm comprising *Streptococcus mutans*, and *Streptococcus gordonii*. 

## MATERIALS AND METHODS

### Materials

*Camellia sinensis* leaves cultivated in Huayopata, Cuzco, Peru. All microbiological processes were performed at the Bacteriology Laboratory of the Cayetano Heredia Peruvian University, using lyophilized strains of *S. mutans* ATCC® 25175 (Microbiologics, USA) and *S. gordonii* ATCC® 51656 (Microbiologics, USA). For positive control, chlorhexidine at 0.12% was used, while dimethyl sulfoxide (DMSO) was used for negative control ^18^. The culture medium used was brain heart infusion (BHI) agar (OXOID, United Kingdom) and filter paper discs (Whatman 2017-006, USA) were used. To measure the inhibition zones, a digital caliper (Mitutoyo, Japan) with a range of 0-150 mm and a precision of 0.01 mm was used.

### Extraction procedure

The organic ethanolic crude extract was prepared using 100 g of dried *Camellia sinensis* leaves mixed with 95 % ethanol and macerating for 24 hours. Then the mixture was filtered using Whatman paper No. 1 (Whatman 2017-006, USA) and evaporated under vacuum (Memmert VOcool 400, Schwabach, Germany) at 40 °C until the complete removal of ethanol, resulting in the crude extract. ^19^^,^^20^ The entire process was conducted in the Biopolymers and Metalopharmaceuticals Research Laboratory at the National University of Engineering (UNI) in Lima, Peru.

### Antibacterial activity determination

To determine the antibacterial activity of *S. mutans* and *S gordonni* individually, a concentration of 25 mg/ml of the crude *Camellia sinensis* extract was used. The antibacterial activity of each strain was identified using the *Kirby-Bauer* agar disk diffusion method. Plates were prepared on BHI agar (OXOID, United Kingdom) controlled for 24 hours to ensure sterility. The strains were cultured in BHI broth (OXOID, United Kingdom) for 24 hours and then brought to the Mac Farland scale of 0.5. The confrontation was performed by soaking a swab with the previously prepared inoculum, then sowing it four times on the surface of the agar, allowing it to stand for 5 minutes, and then placing Whatman filter paper discs n°3, 6 mm in diameter, impregnated with 10 μL of each extract, separately. This procedure was repeated 3 times for each of the groups: G1 crude ethanolic extract of *Camellia sinensis*, G2 positive control 0.12% chlorhexidine (Perio-Aid®-Dentaid, USA), and G3 negative control DMSO (Sigma-Aldrich, USA) plus Milli-Q water (1:1). Then, all plates with extracts and control samples were incubated in anaerobic conditions at 37°C for 48 hours. The reading of the result was made by measuring the diameter of the inhibition halos in mm using a caliper (Mitutoyo, Japan). The sensitivity analysis by means of inhibitions halos were interpreted using the Duraffourd & Lapraz scale, cited by Checallla-Collatupa et al. ^21^ This scale grouped inhibitions into four categories, null (-) for values below 8 mm, limit sensitivity (+) for values between 8 and 14.9 mm, average sensitivity (++) for values between 15 and 20 mm, and high sensitivity (+++) for values greater than 20.1 mm.

### Minimum inhibitory contraction (MIC) determination

Using the broth microdilution method, 96-well microtiter plates were prepared by adding 140 μL of BHI broth to wells 2B through 11B, adding 140 μL of a 50mg/ml of natural extract to well 2B, removing 140 μL with a micropipette from well 2B to well 3B, homogenizing, and repeating the same procedure until well 11B was reached to discard 140 μL. Then, 20 μL of the strain culture was calibrated to the *McFarland* scale of 0.5 was added to wells 2B to 11B and incubated at 37 °C for 48 hours under anaerobic conditions. Chlorhexidine 0.12% (Perio-Aid®-Dentaid, USA) was used as the positive control and DMSO+ Milli-Q® ultrapure (Sigma-Aldrich, USA) as the negative control. The MIC was determined by turbidity analysis of the culture medium. It was considered the concentration of the well where no bacterial growth was observed. In addition, to verify bacterial vitality, 5 μL of each well was seeded on BHI agar plates and incubated for 48 hours at 37 °C under anaerobic conditions. The MIC was determinated by performing a plate reading, as shown in figure 2, where no colony growth was observed. 

### Formation of Multispecies Biofilms 

Using strains of *Streptococcus mutans* ATCC® 25175 and *S. gordonii* ATCC® 51656, a biofilm was formed on the glass surface using the Nunc Lab-Tek Chamber Slide™ system (Thermo Fisher scientific, USA) with 8 wells. The strains were then individually inoculated into 15 ml of BHI agar under anaerobic growth conditions at 37 °C until exponential growth of the bacteria was achieved. *Streptococcus mutans* and *S. gordonii* strains were incubated for 3.5 and 4.5 minutes respectively until optical density values of 0,125 nm with 150 x 106 cells/ml. To cover the wells, 30 µL of poly-L-lysine (SIGMA: 41116105, USA) was used and incubated for 30 minutes. The wells were then washed with 30 µL of phosphate-buffered saline (PBS) (1X) (SIGMA, USA) and dried at 37 °C for 24 hours under aseptic conditions. Three hundred microliters of artificial saliva (Sigma-Aldrich: SAE0149, USA) were added to each well and incubated for 16 hours at 4 °C. The artificial saliva was then removed by washing each well twice with 300 µL of PBS (1X), and 250 μL of BHI broth containing 2.5% sucrose was added. Under anaerobic conditions, 10 μL of *S. mutans* and *S. gordonii* were inoculated into each well and incubated at 37 °C for 24 hours ^22^. 

### Cell viability of Biofilm model

All biofilms were exposed to the ethanolic extract, as well as the positive and negative controls for one minute, every eight hours. Then, they were rinsed twice with 300 µL of PBS (1x) and transferred to fresh sterile culture medium. The biofilms were allowed to grow for 24 hours. Then, 30 µL of the ethanolic extract was administered every eight hours until 96 hours of biofilm formation were reached. This procedure was performed in triplicate with the negative (1% DMSO + Milli-Q water at a 1:1 ratio) and positive (0.12% chlorhexidine) control groups. Evaluations were performed at 24 and 48 hours after biofilm formation. To determine the effect of the *Camellia sinensis* ethanolic extract on *Streptococcus mutans* and *S. gordonii* biofilms, immunofluorescence was performed to evaluate cell vitality. Biofilm cell viability was assessed using immunofluorescence microscopy (Leica DMi8, Wetzlar, Germany) with LIVE/DEAD® BacLight™ staining kits after incubating for 24 and 48 hours. Viable cells appeared green (SYTO 9), and nonviable, membrane-compromised cells appeared red (propidium iodide)

### Statistical analysis

Using STATA 17 software, data were summarized with mean and standard deviation. The normal distribution of the data was determined using the Shapiro-Wilk test (p > 0.05). For the hypothesis contrast of inhibition halo difference between *Camellia sinensis* extract and chlorhexidine, Student’s t-test for independent sample was used, assuming a confidence level of 95% and expected an error of 5%. 

## RESULTS

### Antimicrobial activity of Camellia sinensis

**Figure 1:**
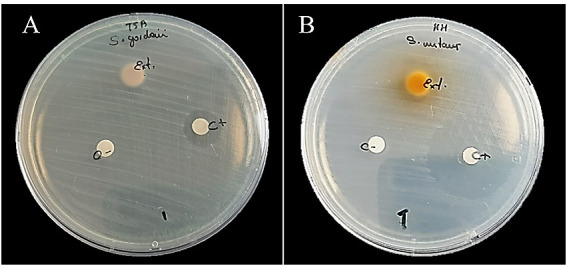
Antibacterial activity with inhibition halos of *Camellia sinensis* crude extract on *Streptococcus mutans* (A) and *Streptococcus gordonii* (B). Extract (Ext.), Chlorhexidine 0.12% (C+), and negative control DMSO+ Milli-Q (C-).

A total of three replicates were analyzed for each group. Similar zones of inhibition were observed within each group, but not between them, as shown in Table 1. The antibacterial activity of *Camellia sinensis*, analyzed by zones of inhibition, is shown in Figure 1 and Table 2. *Camellia sinensis* crude extract inhibited *S. mutans* with an inhibition value of 17.4 mm ± 0.4 mm (95% CI:16.5-18.3 mm), indicating average sensitivity (++). The chlorhexidine group exhibited high sensitivity (+++) with a mean zone inhibition value of 23.6 mm ± 0.3 mm (95% CI: 22.9-24.3 mm). These differences were statistically significant (p<0.001).

**Table 1:** Effect of *Camellia sinensis* organic extract on growth inhibition (mm) of *S. mutans* ATCC 25175 and *S. gordonii* ATCC 51656 strains

Groups	Concentration (mg/ml)	Streptococcus mutans ATCC 25175	Streptococcus gordonii ATCC
*Camellia sinensis*	25 mg/ml	17.3	13.1	17.8	12.8	17.1	13.4
*Positive Control* Clorhexidine	0.12%	23.4	12.8	23.9	13.7	23.5	13.2
*Negative control* DMSO +H2Oq*	1:1 ratio	0.0	0.0	0.0	0.0	0.0	0.0

* Dimethylsulfoxide with Milli-Q (1:1) solution

**Table 2:** Comparison of the inhibitory effect of *Camellia sinensis* extract and control groups on *Streptococcus mutans* and *Streptococcus gordonii* strains

Microorganisms	Groups	Mean±SD	95% CI Mean	Susceptibility±	p_value
*Streptoccocus mutans*	*Camellia sinensis*	17.4±0.4	16.5-18.3	++	<0.001*
	Clorhexidine	23.6±0.3	22.9-24.3	+++	
*Streptococcus gordonii*	*Camellia sinensis*	13.1±0.3	12.4-13.8	+	0.691
	Clorhexidine	13.2±0.5	12.1-14.4	+	

The values represent inhibition zone diameters in mm.

SD (standard deviation)

± Null sensitivity ( ), limited (+), very sensitive (++), highly sensitive (+++).

*Significant differences (p < 0.001)

On the other hand, for *S. gordonii* strain, the inhibitory activity was similar for both the *Camellia sinensis* group, showing borderline sensitivity with inhibitory values of 13.1 mm ± 0.3 mm (95% CI 12.4 mm-13.8 mm), and chlorhexidine with 13.2 mm ± 0.5 mm (95% CI 12.1 mm-14.4 mm), which were not significantly different (p = 0.691).

### Minimum inhibitory concentration (MIC)

The MIC values of *Camellia sinensis* were 3.12 mg/ml for *S. mutans* and 1.56 mg/dl for *S. gordonii*. No bacterial growth was observed at these concentrations (Table 3). 

**Figure 2:**
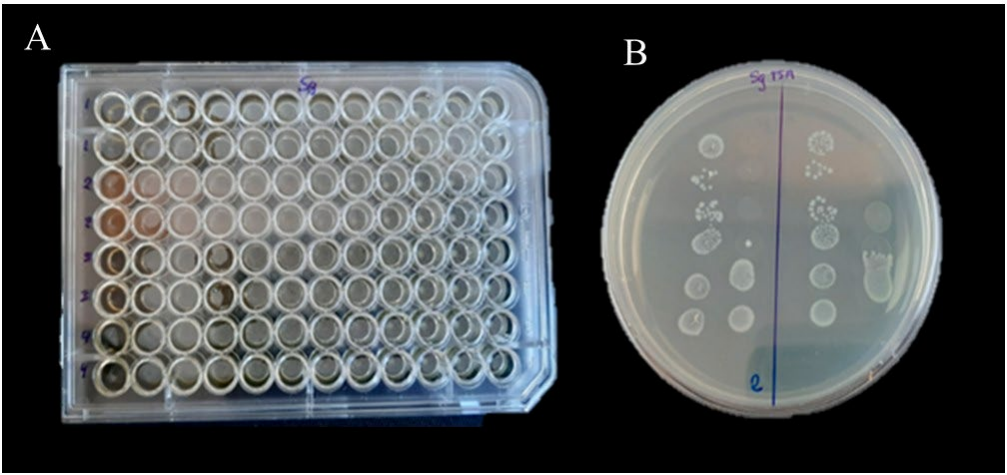
Determination of MIC: (A) 96-well plate with the microdilution method in *S. gordonii* broth. (B) Bacterial growth on *S. gordonii* BHI agar.

**Table 3:** Minimum inhibitory concentration of the crude extract organism of *Camellia sinensis against S.mutans*, *and S. gordonii*

Groups	Concentration (mg/mL)	Streptococcus mutans ATCC 25175	Streptococcus gordonii ATCC 51656
*Camellia sinensis*	50	-	-
	25	-	-
	12.5	-	-
	6.25	-	-
	3.12	-	-
	1.56	+	-
	0.78	+	+
	0.39	+	+
	0.19	+	+
	0.09	+	+
	0.04	+	+
	0.02	+	+
	0.01	+	+
Controls	Clorhexidine 0.12%	-	-
	DMSO + Milli-Q water	+	+

(-) no bacterial growth

(+) bacterial growth

### Cell viability analysis

**Figure 3:**
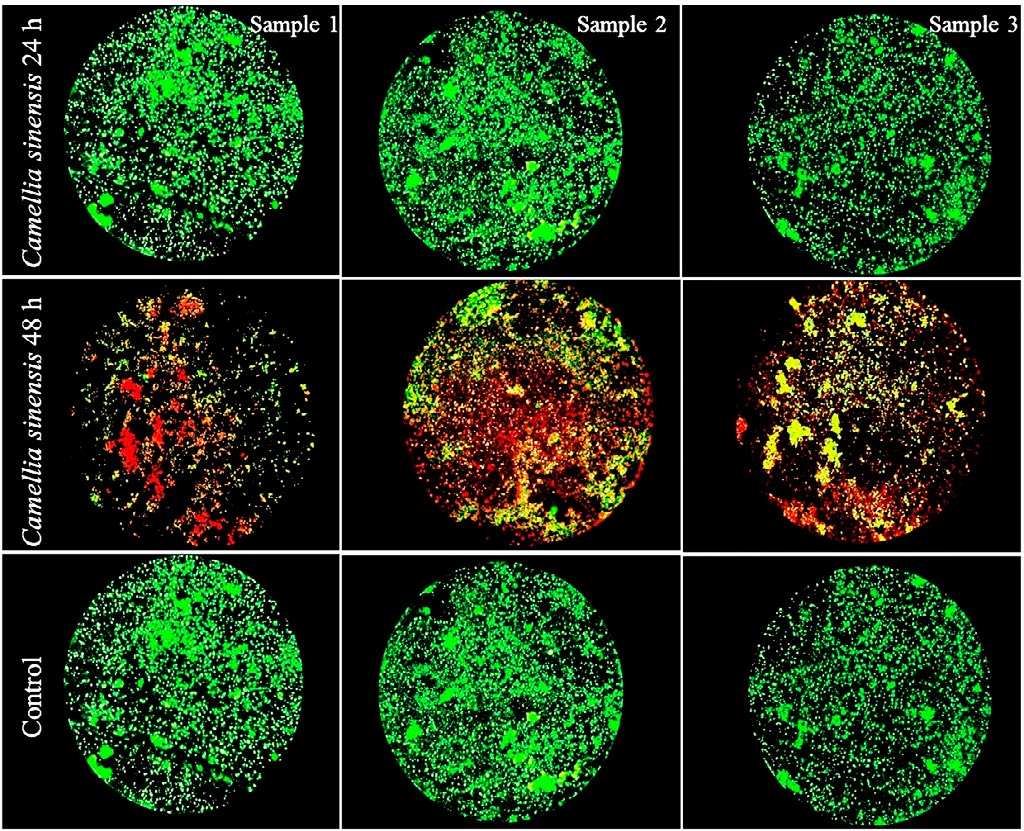
Evaluation of the viability of the *Streptococcus mutans* and *Streptococcus gordonii* bi-species biofilm after 24 h and 48 hours of exposure to green tea extract, compared with the control group. Viable bacteria appear green (SYTO 9), while non-viable bacteria appear red (propidium iodide). Representative images were obtained using fluorescence microscopy (20x magnification).

The anti-biofilm activity of *Camellia sinensis* was assessed using a cell viability analysis. After 24 hours of incubation, high cell viability was observed, with a predominance of green fluorescence predominant in all wells and their respective replicates. This indicates an initial effect on the biofilm architecture without significant alterations to viability in the early stages, as shown in Figure 3. After 48 hours of incubation, the *Camellia sinensis* extract exhibited a stronger antimicrobial effect, with an evident decrease in cell viability. Figure 4 shows a significant increase in red fluorescence in all wells and their respective replicates, indicating greater cell membrane damage and bacterial death. In addition, there was reduced biofilm density, with decreased cohesion and areas of cell dispersion. These results suggest that the crude extract of *Camellia sinensis* has a time-dependent antimicrobial effect that is more effective against biofilms with a longer incubation time.

## DISCUSSION

The high rates of dental caries and periodontal disease worldwide reveal the limited effectiveness of current health strategies against these oral pathologies ^2^^,^^10^. In response to this, phytotherapy has become the ideal alternative, where the study of various plants is leading to the identification of antimicrobial activity and could become one of the main therapies in the coming years ^8^^,^^23^. 

In this context, the various extracts of *Camellia sinensis* are presented as an alternative that could have a potentiating effect against bacterial agents acting on biofilms formed by multiple species, especially *S. mutans*, a species related to the onset and progression of dental caries, and *S. gordonii* related to the formation of the dental plaque and apical periodontitis ^24^^-^^26^. 

The antibacterial effect against some oral bacteria is explained by its high concentration of polyphenols, specifically the group of catechins considered as agents with caries control potential. The activity of the different catechins ranges from effects on growth, aggregation, and acid production of *S. mutans*^15^^,^^27^^,^^28^. 

In this study, the analysis was limited to the organic crude extract of *Camellia sinensis* leaves. The crude extract was chosen because it contains the greatest diversity of bioactive polyphenolic compounds, which are responsible for the antibacterial activity of extracts. This enables us to conduct a more comprehensive initial assessment of its inhibitory potential while avoiding the loss of synergy between the active components that can occur during fractionation processes ^29^^,^^30^. A phytochemical analysis was not performed, considering the same composition as evidenced in the scientific literature. Environmental and cultivation conditions could affect the composition and concentration of polyphenols, particularly catechins, responsible for the antimicrobial activity of *Camellia sinensis* against *S. mutans*^15^^,^^27^. In addition, a single concentration of 25 mg/dl extract was used, considered as a high concentration to identify activity without water or alcohol.

The crude ethanolic extract of *Camellia sinensis* showed significant antibacterial activity against *Streptococcus mutans* and *Streptococcus gordonii*, species that are representative of the initial cariogenic microbiota. The extract produced inhibition zones of 17.4 ± 0.4 mm for *S. mutans* and 13.1 ± 0.3 mm for *S. gordonii*, values that indicate moderate and limited susceptibility, respectively, compared with those obtained with chlorhexidine (23.6 ± 0.3 mm and 13.2 ± 0.5 mm). Although chlorhexidine maintained greater potency against *S. mutans*, both substances showed comparable efficacy against *S. gordonii*, which suggests that the phenolic compounds of green tea could play a relevant role in inhibiting primary colonizers during the early stages of biofilm formation. These results agree with some reports that conducted experiments using different hydroalcoholic or aqueous extracts comparing them with chlorhexidine. In addition, the studies were conducted with plants grown mostly in the Asian region ^13^^,^^31^^-^^33^. Moreover, the study by Zayed et al. reported activity with bacterial inhibition against *S. mutans* but with a wide MIC range from 3.1 to 12.5 mg/dl, contrasting with our study that found an MIC value of 3.12 mg/dl for *S. mutans*, and 1.56 mg/dl for *S. gordoni*^30^. The MIC values fall within the range reported in previous studies conducted with ethanolic and hydroalcoholic extracts of *Camellia sinensis*. These similarities confirm the reproducibility of the plant’s antimicrobial effect, although it is important to consider that factors such as geographic origin, growing conditions, and extraction method can modify the phytochemical profile and, therefore, the biological potency of the extract. 

Likewise, Anita et al. reported activity against *S. mutans* with an average zone of inhibition of 14.67 mm for the hydroalcoholic extract of dried *Camellia sinensis* leaves ^31^. Nevertheless, it is important to highlight the low concentrations of 100, 200 and 300 ug used in the study with inhibition levels ranging from sensitive to very sensitive, aligning to our findings. This would indicate that the effectiveness against this bacterium is independent of the type of extract used. However, it is necessary to conduct comparative studies including concentrations with activity demonstrated by previous studies.

Along the same lines but using a different *Camellia sinensis* preparation, in their study, Goto et al. ^13^, reveal a strong inhibitory activity on the biofilm formation of *S. mutans*. *Camellia sinensis* powders from leaves subjected to high temperatures were used to improve their odor and flavor properties. This approach allowed us to observe not only direct antibacterial action, but also possible alterations in the structure and cohesion of the biofilm, evidenced by the decrease in density and the loss of cellular integrity detected by immunofluorescence after 48 hours. These findings suggest that the *Camellia sinensis* extract could act by modulating biofilm stability and limiting its maturation rather than by eliminating cells immediately.

Other forms of *Camellia sinensis* inclusion have also been used to assess its antibacterial activity, as reported by Moein et al. ^33^, who used a commercial mouthwash containing *Camellia sinensis* extract as one of its main components. The results of this study show a high antibacterial activity of the mouthwash containing *Camellia sinensis* extract, with a highly sensitive inhibition for *S. mutans*. However, it is not possible to conclude the specific contribution of *Camellia sinensis*, as the mouthwash used contains alcohol, among other components with potential inhibitory effects against various microorganisms.

None of the studies analyzed included *S. gordonii* to evaluate the inhibitory activity of the crude organic *Camellia sinensis* extract. Therefore, our study is the first to analyze its effect on this organism. This is particularly important in the context of periodontal diseases with periapical lesions ^27^^,^^34^. Moreover, this microorganism has been reported to have the ability to spread throughout the bloodstream, becoming a causal agent of various pathologies such as periapical infections, septic arthritis, bacterial peritonitis, necrotizing pneumonia, and in some cases, bacterial endocarditis ^35^. 

Among the limitations of the study, we recognize the absence of phytochemical characterization of the extract, the use of a single concentration, and the in vitro nature of the experimental model, which does not fully reproduce the physiological conditions of the oral environment. However, the results obtained provide solid evidence of the antimicrobial potential of green tea and justify future research aimed at evaluating fractionated extracts, identifying the active compounds responsible, and analyzing possible synergistic effects with conventional antimicrobials such as chlorhexidine.

New hypotheses are generated from these results including the comparative effect of different concentrations, formulations, and extracts, as well as from different regions, against other oral bacteria with cariogenic potential and oral biofilm formation. Additionally, the comparative analysis of the phytochemical composition in plants cultivated in different regions, assuming that environmental and cultivation conditions could influence the composition and concentrations of key elements related to some type of activity against various microorganisms.

Likewise, the evaluation of formulations with different vehicles or delivery systems could help improve the bioavailability and stability of the active compounds in biological media, increasing their applicability in oral hygiene products. If its efficacy is confirmed in clinical models, the *Camellia sinensis* extract could constitute a natural and less cytotoxic alternative for biofilm control and the prevention of oral pathologies.

## CONCLUSION

Within the limitations of the present in vitro study, the crude ethanolic extract of *Camellia sinensis* demonstrated antibacterial activity against *Streptococcus mutans* and *Streptococcus gordonii*, with efficacy comparable to chlorhexidine against the latter species. In addition, a progressive inhibitory effect on the cell viability of the bi-species biofilm was observed after 48 hours of exposure, confirming its time-dependent antimicrobial action. These results support the potential of *Camellia sinensis* as a natural alternative or complementary agent in the control of cariogenic biofilm, especially during the initial stages of its formation. Additional studies are recommended, including phytochemical characterization of the extract, the evaluation of clinically relevant doses, and its validation in in vivo models to determine its applicability in therapeutic or preventive formulations intended for oral care.
